# Activation of Gold on Metal Carbides: Novel Catalysts for C1 Chemistry

**DOI:** 10.3389/fchem.2019.00875

**Published:** 2020-01-08

**Authors:** José A. Rodriguez

**Affiliations:** Brookhaven National Laboratory, Department of Chemistry, Upton, NY, United States

**Keywords:** gold, metal carbides, C1 chemistry, water-gas shift reaction, CO_2_ hydrogenation

## Abstract

This article presents a review of recent uses of Au-carbide interfaces as catalysts for C1 Chemistry (CO oxidation, low-temperature water-gas shift, and CO_2_ hydrogenation). The results of density-functional calculations and photoemission point to important electronic perturbations when small two-dimensional clusters of gold are bounded to the (001) surface of various transition metal carbides (TiC, ZrC, VC, Ta C, and δ-MoC). On these surfaces, the C sites exhibited strong interactions with the gold clusters. On the carbide surfaces, the Au interacts stronger than on oxides opening the door for strong metal-support interactions. So far, most of the experimental studies with well-defined systems have been focused on the Au/TiC, Au/δ-MoC, and Au/β-Mo_2_C interfaces. Au/TiC and Au/δ-MoC are active and stable catalysts for the low-temperature water-gas shift reaction and for the hydrogenation of CO_2_ to methanol or CO. Variations in the behavior of the Au/δ-MoC and Au/β-Mo_2_C systems clearly show the strong effect of the metal/carbon ratio on the performance of the carbide catalysts. This parameter substantially impacts the chemical behavior of the carbide and its interaction with supported metals, up to the point of modifying the reaction rate and mechanism of C1 processes.

## Introduction

In the last 15 years, several studies have shown that Au nanoparticles dispersed on carbide surfaces can be very active as catalysts for process related to C1 chemistry such as the oxidation of carbon monoxide (CO + 0.5O_2_ → CO_2_) (Ono et al., 2006; Rodriguez et al., 2010), the water-gas shift reaction (CO + H_2_O → H_2_ + CO_2_) (Rodriguez et al., 2014; Posada-Perez et al., 2017; Yao et al., 2017), and the hydrogenation of carbon dioxide to methanol (CO_2_ + 3H_2_ → CH_3_OH + H_2_O) (Vidal et al., 2012; Rodriguez et al., 2013; Posada-Pérez et al., 2016). It is quite interesting that Au is activated by bonding interactions with carbide substrates. For many years, a lot of attention has been focused on examining the properties of Au in contact with different types of oxide supports (Al_2_O_3_, MgO, CeO_2_, TiO_2_, InO_2_, ZrO_2_, CrO_x_, MnO_x_, Fe_2_O_3_) (Haruta, 1997; Fu et al., 2003; Campbell, 2004; Zhang et al., 2005; Yang et al., 2013; Gu et al., 2014). Bulk metallic Au displays a low reactivity as a consequence of combining a deep-lying valence d band and very diffuse valence s, p orbitals (Hammer and Nørskov, 1995). In the literature, the activation of supported gold has been explained using several models: From special chemical properties resulting from the limited size of the active gold particles (usually <5 nm), to the effects of charge transfer between the oxide and gold. What happens when gold is dispersed on a substrate which has physical and chemical properties different from those typical of an oxide? The carbides of the early-transition metals have a much lower ionicity than typical oxides and exhibit, in many aspects, a chemical behavior similar to that of noble metals (Hwu and Chen, 2005).

**Figure 1:**
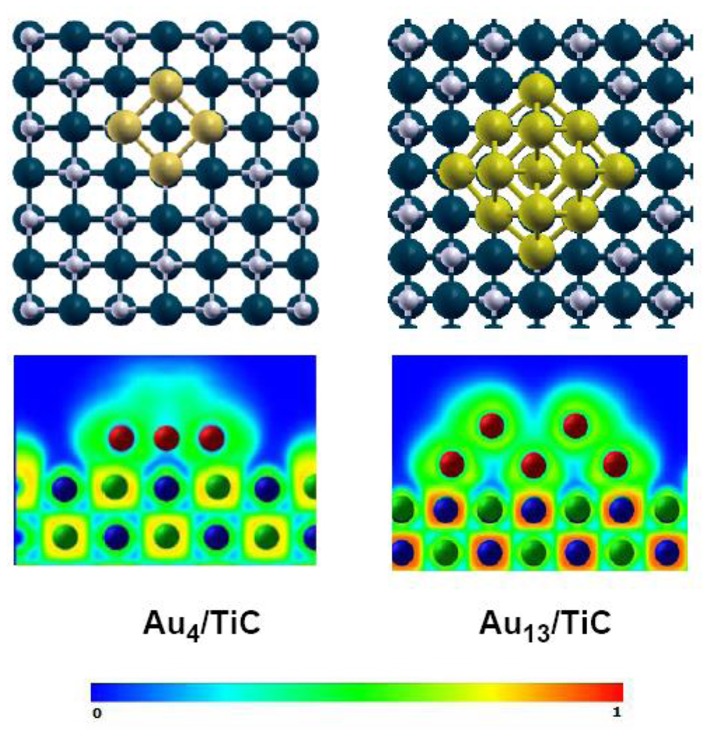
Bonding configurations (top section) and ELF maps (bottom section) calculated for Au_4_ and Au_13_ clusters on TiC(001) using DF-based methods. Color code: Carbon atoms are represented by gray spheres, titanium atoms as blue spheres, and gold atoms as yellow spheres. The Au_13_ cluster contains two layers of 9 and 4 metal atoms. Bottom, ELF maps: The probability of finding an electron varies from 0 (blue color) to 1 (red color). Reproduced with permission from Rodriguez and Illas (2012), copyright 2012 by the Royal Society of Chemistry.

The inclusion of C into the lattice of an early-transition metal modifies the chemical reactivity of the system through ensemble and ligand effects (Liu and Rodriguez, 2004; Hwu and Chen, 2005; Rodriguez and Illas, 2012). After forming a compound, the presence of the carbon atoms in the lattice puts a limit in the total number of metal atoms that can be present in a surface of a metal carbide (ensemble effect). Furthermore, the formation of metal-carbon bonds perturbs the electronic properties of the metal (reduction in its density of states near the Fermi level; a net metal → carbon charge transfer) (Liu and Rodriguez, 2004; Hwu and Chen, 2005), making it less chemically active (ligand effect) and a better catalyst according to the Sabatier's principle (Liu and Rodriguez, 2004). The electron-rich carbon atoms present in carbide surfaces interact well with Au adatoms (Rodriguez and Illas, 2012). A charge polarization induced by Au↔C interactions (Figure 1) produces systems which exhibit a chemical activity much larger than those found after the deposition of gold on surfaces of oxides (Rodriguez and Illas, 2012).

In this article, a short review on the uses Au-carbide interfaces in C1 catalysis is presented. The text is organized as follows. The next section describes studies dealing with CO oxidation (Ono et al., 2006; Rodriguez et al., 2010). Then, we focus on works examining the water-gas shift reaction (Rodriguez et al., 2014; Posada-Perez et al., 2017; Yao et al., 2017). This is followed by studies on the activation of CO_2_ and its conversion into CO or methanol (Vidal et al., 2012; Rodriguez et al., 2013; Posada-Pérez et al., 2016). The article ends with a discussion on future directions for the use of Au-carbide interfaces in C1 catalysis.

## CO Oxidation

Roldan-Cuenya et al. studied the growth mode of Au on TiC films using scanning microscopy (STM) (Naitabdi et al., 2006; Ono et al., 2006; Ono and Roldan-Cuenya, 2007). In general, the gold did not wet well the carbide surface. It formed three-dimensional (3D) nanoparticles at medium and large coverages. Measurements of scanning tunneling spectroscopy (STS) showed the existence of a band gap for the Au nanoparticles with heights in the range of 1.3 to 2.1 nm. The Au/TiC systems were able to perform the low-temperature oxidation of CO (Ono et al., 2006; Ono and Roldan-Cuenya, 2007). This motivated a detailed study of the interaction of Au with TiC(001) (Rodriguez et al., 2007). STM images and XPS data for Au on TiC(001) point to a lack of layer-by-layer growth, with the admetal forming 2D and 3D islands over the carbide surface (Rodriguez et al., 2007). High-resolution photoemission data point to a strong Au↔TiC(001) interaction (Rodriguez et al., 2007). The C 1s photoemission results indicate that Au prefers to interact with the carbon centers of TiC(001). Density functional (DF) calculations for the bonding of gold atoms and a series of clusters (Au_2_, Au_4_, Au_13_, Au_29_) on TiC(001) also give preferential adsorption on C sites (Rodriguez et al., 2007). Figure 1 shows calculated electron-localization function (ELF) (Silvi and Savin, 1994) plots for clusters of Au_4_ and Au_13_ bonded to TiC(001). For the Au_4_/TiC(001) system, one can see a substantial accumulation of electrons in the region outside the Au_4_ cluster. A phenomenon which was also seen when Au, Au_2_, and other small clusters where deposited on the carbide substrate (Rodriguez et al., 2007). In the case of Au_13_/TiC(001), the gold cluster now has two layers, with gold atoms that are not in contact with the support. These second-layer atoms, as shown in Figure 1, do not exhibit a polarization of electrons as pronounced as found in the case of Au_4_/TiC(001). In fact, for Au_13_/TiC(001), the polarization of electrons in the first layer is minor. The DF results in Figure 1 are consistent with results of photoemission which point to electronic perturbations on gold only at small coverages of the metal (Rodriguez et al., 2007). Theory and experiment show that one really needs small 2D gold clusters in contact with TiC(001). The results of several theoretical studies dealing with metal-carbide interfaces predict big differences between the chemical reactivity of 2D and 3D gold clusters (Zhang et al., 2005). A phenomenon which have been experimentally verified for several catalytic processes (Vidal et al., 2012; Rodriguez et al., 2013, 2014; Posada-Pérez et al., 2016; Posada-Perez et al., 2017; Yao et al., 2017).

**Figure 2:**
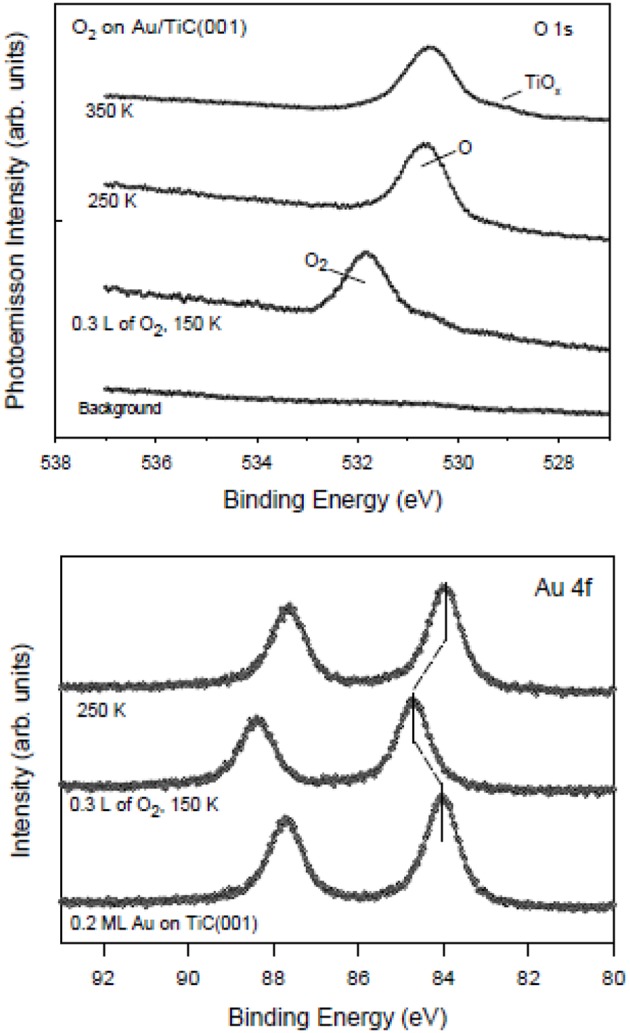
Results of XPS, O 1s (top panel) and Au 4f regions (bottom panel), collected after dosing O_2_ to a TiC(001) substrate with 0.2 ML of gold. The initial dosing of molecular O_2_ was done at 150 K. Then, the O_2_/Au/TiC(001) surface was annealed to 250 and 350 K. Reproduced with permission from Rodriguez et al. (2010), copyright 2010 by the American Society of Chemistry.

Nanoparticles of gold dispersed on TiC films and TiC(001) oxidize carbon monoxide (2CO + O_2_ → 2CO_2_) at temperatures below 200 K (Ono et al., 2006; Rodriguez et al., 2010). Following the coadsorption of CO and O_2_ at ~100 K, the evolution of CO_2_ was detected at 160–180 K during the ramping up of the temperature (Ono et al., 2006; Rodriguez et al., 2010). Neither pure metallic gold nor regular TiC promote the low temperature oxidation of CO. Thus, the oxidation of CO is probably occurring on the small Au particles or on the gold-carbide interface. DF calculations predict similar adsorption energies for CO on TiC(001) and Au/TiC(001) surfaces (Rodriguez et al., 2010). Thus, the good performance of the Au/TiC system in CO oxidation must be a direct result of the ability that the Au in contact with TiC has to activate the O_2_ molecule (Rodriguez et al., 2010). The calculated O_2_ adsorption energy varies from −0.45 eV on TiC(001) to −1.41 eV on Au_4_/TiC(001) (Rodriguez et al., 2010). Furthermore, the O–O bond length rises from 1.23 Å in free O_2_ to 1.55 Å in adsorbed O_2_. On Au_4_/TiC(001), the O–O bond is not broken, but the O_2_ molecule has been activated and can react with CO molecules initially bound to TiC(001) surface or directly attached to gold (Rodriguez et al., 2010). The photoemission results displayed in Figure 2 indicate that Au/TiC(001) interacts well with O_2_. At 150 K, O_2_ is chemisorbed but at higher temperatures it dissociates to produce O adatoms that can react with CO in an oxidation process (Rodriguez et al., 2010).

## Water-gas Shift Reaction

**Figure 3:**
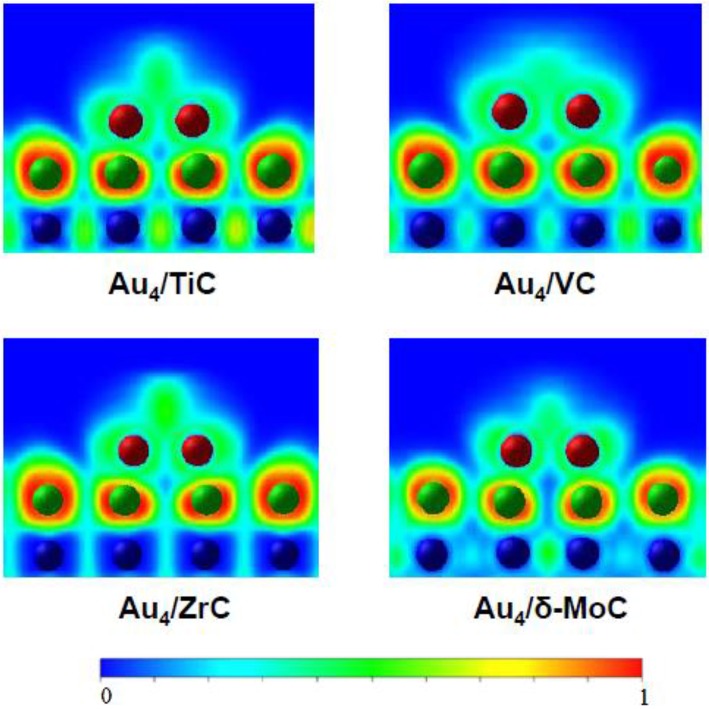
Calculated electron densities for a Au_4_ cluster on different carbide substrates. Reproduced with permission from Florez et al. (2009), copyright 2009 by the American Chemical Society.

Gold nanoparticles dispersed on TiC, MoC, and Mo_2_C display high activity for the low temperature water-gas shift (LT-WGS) reaction (Rodriguez et al., 2014; Posada-Perez et al., 2017; Yao et al., 2017). As mentioned above, and shown in Figure 1, small clusters of gold in direct contact with TiC(001) exhibit important electronic perturbations (Rodriguez et al., 2007). A similar phenomenon has been found after depositing the noble metal on surfaces of carbides of molybdenum and other metals. DF calculations were performed to examine in a systematic way the electronic structure of a series of small gold clusters (Au_2_, Au_4_, Au_9_, Au_13_, and Au_14_) bounded to the (001) surface of various transition metal carbides (δ-MoC, TiC, VC, and ZrC) (Florez et al., 2009). On these surfaces, the C sites exhibited strong interactions with the gold clusters. Bonding to the atoms of the underlying carbide strongly modified the electronic structure and charge density of the bound metal clusters. For 2D gold systems in direct contact with the carbide substrates, the electronic perturbations were quite strong (see Figure 3), but they gradually decreased when going to two-layer and three-layer gold systems. In general, the results of the DF calculations suggest that Au atoms in contact with carbide surfaces could be catalytically active (Florez et al., 2009). For Au/TiC and Au/MoC, this prediction has been verified at an experimental level for the LT-WGS reaction (Rodriguez et al., 2014; Posada-Perez et al., 2017; Yao et al., 2017).

**Figure 4:**
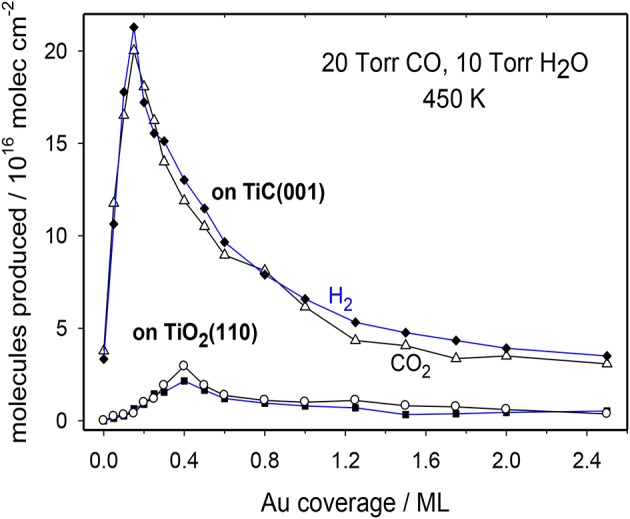
Catalytic activity for the LT-WGS on Au/TiC(001) and Au/TiO_2_(110) surfaces as a function of Au coverage. T = 450 K, 10 Torr of H_2_O and 20 Torr of CO. Reproduced with permission from Rodriguez et al. (2014), copyright 2014 by Wiley.

The WGS activity for plain TiC(001) and Au/TiC(001) systems with a broad set of gold coverages is shown in Figure 4 (Rodriguez et al., 2014). The clean TiC(001) is a catalyst for the water-gas shift. Interestingly, at a temperature of 450 K, TiC(001) has a WGS activity larger than that of Cu(111) (Nakamura et al., 1990), which is a common benchmark in WGS studies (Nakamura et al., 1990; Gokhale et al., 2008). Extended surfaces of metallic Au are not able to catalyze the WGS process (Si et al., 2012). In spite of this, the addition of gold to a TiC(001) surface largely enhances the WGS activity of the system. A maximum for the generation CO_2_ and H_2_ is detected at a Au coverage of ~0.15 ML. Beyond this coverage, the WGS activity of Au/TiC(001) gradually decreases (Rodriguez et al., 2014). Images of STM indicate that at coverages below 0.2 ML, Au grows on TiC(001) forming a large amount of 2D particles where the gold atoms are bonded to the C sites of the substrate and undergo an electron polarization which increases their chemical reactivity (Rodriguez et al., 2007, 2014; Rodriguez and Illas, 2012). At Au coverages above 0.2 ML, the admetal prefers the formation of 3D particles (i.e., a big fraction of the gold atoms are not in contact with the carbide support and do not have special chemical properties).

**Figure 5:**
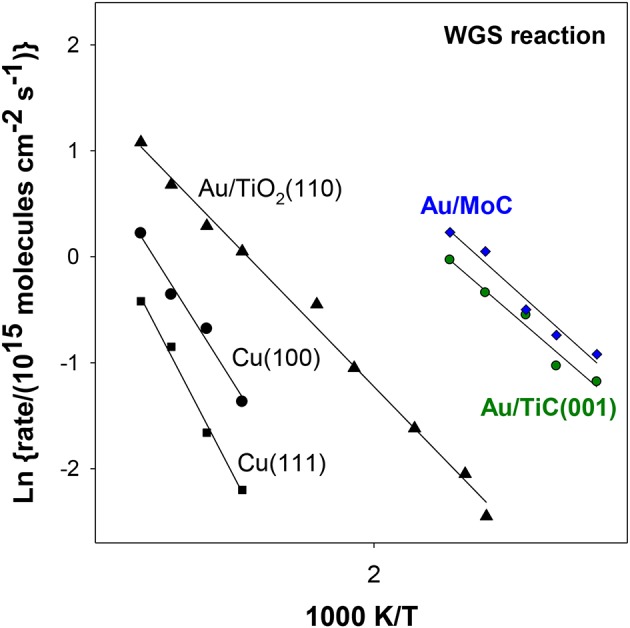
Arrhenius plots for the LT-WGS reaction over copper, Au/oxide and Au/carbide surfaces. Reaction conditions: 10 Torr of H_2_O and 20 Torr of CO. Data taken from Si et al. (2012), Rodriguez et al. (2014), and Posada-Perez et al. (2017).

In Figure 4, the WGS activity of Au/TiC(001) and Au/TiO_2_(110) catalysts with similar amounts of the admetal is compared (Rodriguez et al., 2014). In the range of 550–625 K, Au/TiO_2_ is an excellent catalyst for the WGS exhibiting a higher activity than that seen for Cu/ZnO which is a common industrial WGS catalyst (Si et al., 2012). However, at 450 K, the data in Figure 4 indicate that Au/TiC(001) is the superior low-temperature WGS catalyst. This is corroborated by the results displayed in the Arrhenius graph of Figure 5 where the apparent activation energy for the WGS process drops from 18 kcal/mol on Cu(111) to 10 kcal/mol on Au/TiO_2_(110) and 8 kcal/mol on Au/TiC(001). At temperatures below 500 K, the Au/TiC(001) system has a WGS activity which is observed on pure Cu surfaces and on Cu/oxide or Au/oxide (oxide = TiO_2_, ZnO, CeO_2_, MgO) catalysts only at elevated temperatures (>550 K) (Burch, 2006; Rodriguez et al., 2014). At low temperatures the active sites of the oxide-based systems are usually poisoned by carbonate and formate species. These species have a limited stability on carbide-based catalysts (Rodriguez et al., 2014; Posada-Perez et al., 2017; Yao et al., 2017).

**Figure 6:**
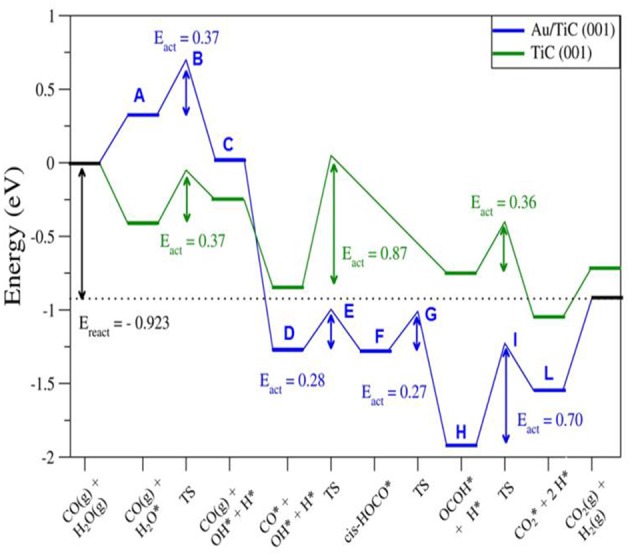
Calculated reaction profiles for the water-gas shift reaction on TiC(001) and Au/TiC(001). The structures for the different intermediates and transition states (A-L) are displayed in Figure 7. Reproduced with permission from Rodriguez et al. (2014), copyright 2014 by Wiley.

**Figure 7:**
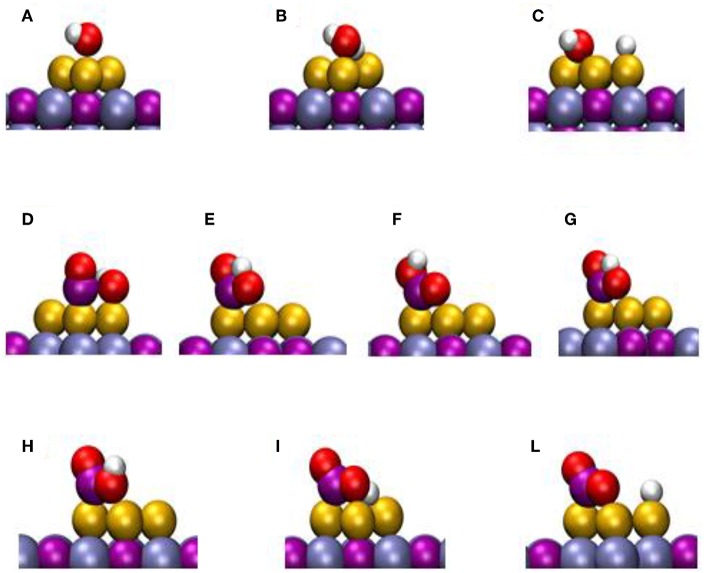
Calculated structures for different intermediates of the WTS reaction on Au/TiC(001). The labels refer to specify states in Figure 6. Reproduced with permission from Rodriguez et al. (2014), copyright 2014 by Wiley.

DF calculations were used to determine the corresponding reaction profiles for the WGS on clean TiC(001) and a Au/TiC(001) catalyst, see Figure 6 (Rodriguez et al., 2014). Figure 7 shows the calculated geometries for the reaction intermediates and the corresponding transition states. A Au_4_/TiC(001) model was used to represent the catalyst. Such a model contains the electronic perturbations produced by bonding gold to the carbide (Figure 1) and reflects the high activity seen for very small coverages of gold on titanium carbide (Rodriguez et al., 2014). In Figure 7, the essential steps for the WGS reaction occur on the gold sites. The DF calculations indicate that on Au/TiC(001) the most favorable path for the WGS involves an associative mechanism where a HOCO species is generated by the interaction of CO with an OH group produced by the dissociation of adsorbed H_2_O. The existence of a key HOCO intermediate, which decomposes into CO_2_ and H, also has been proposed on many metal and metal/oxide catalysts (Burch, 2006; Gokhale et al., 2008). Over TiC(001), the rate constant calculated for the OH + CO➜cis-HOCO reaction was only 3.20 s^−1^ site^−1^, while the corresponding rate was 4.14 × 10^8^ s^−1^ site^−1^ over Au_4_/TiC(001) (Rodriguez et al., 2014). The surfaces of metallic gold do not cleave the O-H bonds of water. In contrast, the calculated rate constant for the dissociation of H_2_O over a Au_4_ aggregate deposited on TiC(001) was 2.02 × 10^9^ s^−1^ site^−1^ (Rodriguez et al., 2014). The fast dissociation of water and the fast formation of the HOCO lead to a high catalytic activity for Au/TiC.

**Figure 8:**
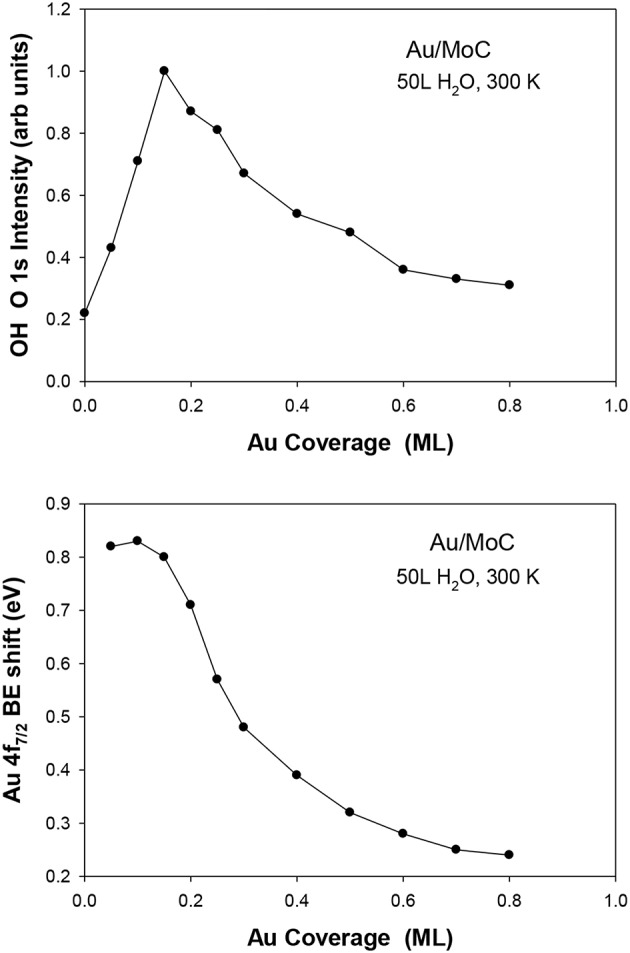
Results of XPS collected after dosing 50 langmuir (L) of water at 300 K to δ-MoC and Au/δ-MoC surfaces. Top: Amount of OH seen in the O 1s region. Bottom: Corresponding binding shift in the Au 4f_7/2_ binding energy. Reproduced with permission from Posada-Perez et al. (2017), copyright 2017 by Royal Society of Chemistry.

The deposition of gold on different surfaces of molybdenum carbide also produces excellent catalysts for the LT-WGS reaction (Posada-Perez et al., 2017; Yao et al., 2017). In Figure 5, the Au/MoC system displays a somewhat better activity than Au/TiC(001) when tested under similar reaction conditions (Rodriguez et al., 2014; Posada-Perez et al., 2017). In the case of Au/MoC, experimental studies do show a clear correlation between the ability of the system to dissociate water and its LT-WGS activity (Posada-Perez et al., 2017). In Figure 8, the amount of OH groups deposited on the surface upon interaction with water increased when small coverages of Au (<0.25 ML) were deposited on MoC. These Au/MoC surfaces displayed a very high activity for the LT-WGS reaction. In Au 4f XPS spectra, there was a binding energy shift that is consistent with the direct dissociation of water on the supported gold. At large coverages of Au (>0.25 ML), 3D particles were formed diminishing the interaction of Au atoms with the carbide substrate (Posada-Perez et al., 2017). As a result of this, the ability of the Au/MoC to dissociate water and catalyze the LT-WGS process diminished (Posada-Perez et al., 2017). In the Au/MoC and Au/TiC(001) systems, the highest catalytic activity is found at very low coverages of gold.

**Figure 9:**
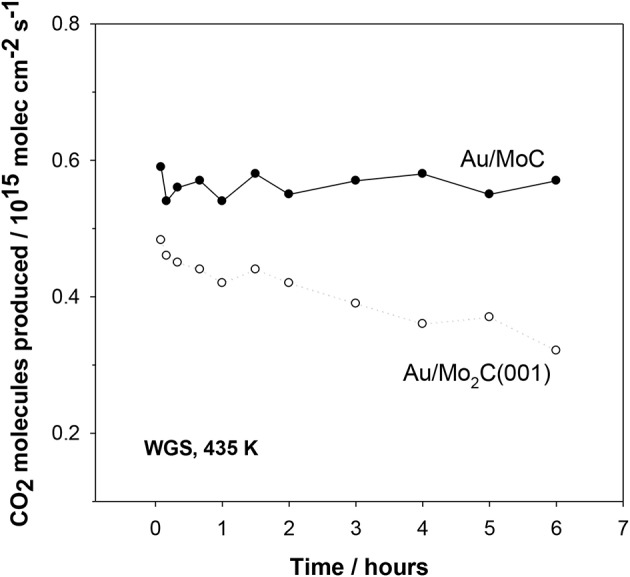
Effect of time on the water-gas shift activity of Au/δ-MoC and Au/β-Mo_2_C(001) surfaces. Initially, 0.15 ML of Au were deposited on the carbide substrates and the obtained catalysts were exposed to 20 Torr of CO and 10 Torr of H_2_O at 435 K. For the Au/δ-MoC system, the coverage of oxygen found after reaction with XPS remained constant (~0.25 ML). Such was not the case for the Au/Mo_2_C(001) system, where a substantial coverage of oxygen was always present (>0.5 ML) and rised with time causing the deactivation of the system. Reproduced with permission from Posada-Perez et al. (2017), copyright 2017 by Royal Society of Chemistry.

Figure 9 compares the stability of Au/MC and Au/Mo_2_C(001) catalysts (Posada-Perez et al., 2017). No signs of deactivation are observed for the Au/MoC system but there is a clear drop in the catalytic activity of Au/Mo_2_C(001). The Mo_2_C is very aggressive toward the oxygen present in the water molecule. Eventually Mo_2_C is transformed into an oxycarbide and the Au↔carbide interactions disappear with a continuous drop in catalytic activity (Posada-Perez et al., 2017). Thus, an important parameter to consider when designing Au/carbide catalysts is the metal/carbon ratio in the carbide support (Rodriguez et al., 2010). This ratio is extremely important because it conditions the reactivity of the metal component in the carbide (Liu and Rodriguez, 2004; Hwu and Chen, 2005). In the case of TiC and MoC, the high concentration of C diminishes the reactivity of the metal centers toward O-containing molecules and at the same time C atoms help to activate the supported Au (Rodriguez and Illas, 2012).

A novel synthetic procedure was used to synthesize atomic-layered Au clusters on a α-MoC substrate (Yao et al., 2017). Images for the Au/α-MoC catalyst obtained using aberration-corrected scanning transmission electron microscopy (STEM) analysis showed that the catalyst structure contained porous assemblies of small α-MoC nanoparticles with a size in the range of 3 to 20 nm and rich in defects. High-resolution STEM *Z*-contrast imaging showed two kinds of gold species on the surface of the catalyst: (i) small layered gold aggregates epitaxially grown on the α-MoC substrate and (ii) atomically dispersed gold (Yao et al., 2017). The gold aggregates had an average diameter of 1 to 2 nm with a thickness of 2 to 4 atomic layers (<1 nm). Catalytic tests showed that both types of supported gold systems were catalytically active but the small layered Au clusters were the most active as catalysts for the LT-WGS process (Yao et al., 2017).

**Figure 10:**
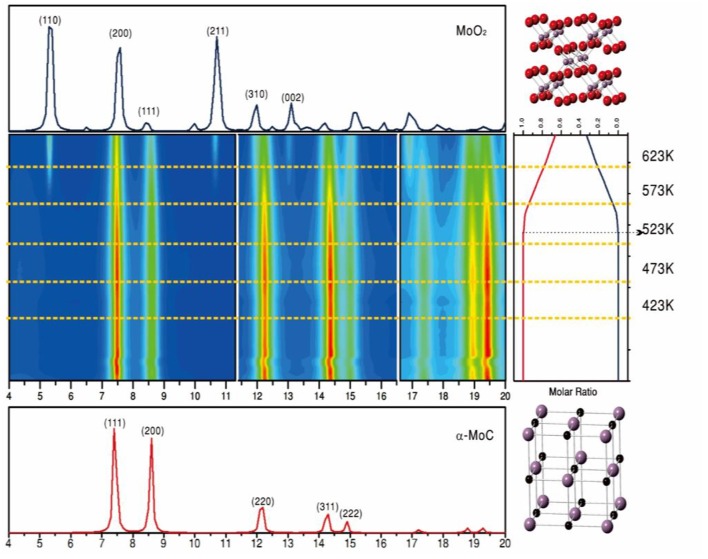
*In situ* time-resolved X-ray diffraction patterns collected for a (2%)Au/α-MoC powder catalyst at various temperatures under normal WGS reaction conditions (wavelength, 0.3196 Å). In the middle panel, the rainbow color scheme varies from no signal (blue) to very intense diffraction peaks (red). The crystal structures of α-MoC and MoO_2_ are shown on the right side of the figure as ball-and-stick drawings (red, O; purple, Mo; black, C). Reproduced with permission from Yao et al. (2017), copyright 2017 by AAA Science.

Results of ambient-pressure XPS showed dissociation of H_2_O over the α-MoC component at room temperature, while the CO was bound to adjacent gold sites. This CO readily reacted with the surface OH groups formed from water, leading to a large LT-WGS activity (390–476 K temperature range) (Yao et al., 2017). At 473 K, the Au/α-MoC catalysts exhibited some deactivation but after testing over a period of 140 h the performance of the catalysts was stable with a CO conversion close to 50% (Yao et al., 2017). A performance which was better than those of Au/oxide catalysts under the same conditions (Yao et al., 2017). The Au/α-MoC catalysts were not stable when exposed to the reactants of the WGS at high temperatures. Results of *in-situ* X-ray diffraction, Figure 10, showed a progressive transformation of α-MoC into MoO_2_ at temperatures above 500 K (Yao et al., 2017). The lack of stability got worse when α-MoC was replaced with ß-Mo_2_C (Yao et al., 2017). Thus, two important parameters to consider when dealing with the long term performance of these systems are the temperature and metal/carbon ratio in the carbide component of the catalysts (Posada-Perez et al., 2017; Yao et al., 2017).

## CO_2_ Hydrogenation to Methanol and CO

Carbon dioxide does not interact with gold at all, but when nanoparticles of the noble metal are deposited on surfaces of carbides, one obtains very good catalysts for the conversion of CO_2_ to methanol or CO (Vidal et al., 2012; Posada-Pérez et al., 2016). In general, the carbides by themselves are active catalysts for the conversion of CO_2_ (Dubois et al., 1992; Xu et al., 2014). Depending on the metal/carbon ratio in the carbide, the products of the of the CO_2_ hydrogenation reaction vary from CO to alcohols and to light alkanes (Dubois et al., 1992; Xu et al., 2014). When the metal/carbon ratio is close to one, CO and methanol are the main products for the hydrogenation of CO_2_ over a carbide catalyst (Dubois et al., 1992; Xu et al., 2014; Posada-Pérez et al., 2016). Theoretical calculations have shown that, in general, CO_2_ binds well on MC(001) surfaces (M = Ti, Mo, Zr, Hf, Nb, Ta, Hf, and W) (Vidal et al., 2012; Posada-Perez et al., 2014; Posada-Pérez et al., 2016; Kunkel et al., 2016; Dixit et al., 2017; Koverga et al., 2019).

**Figure 11:**
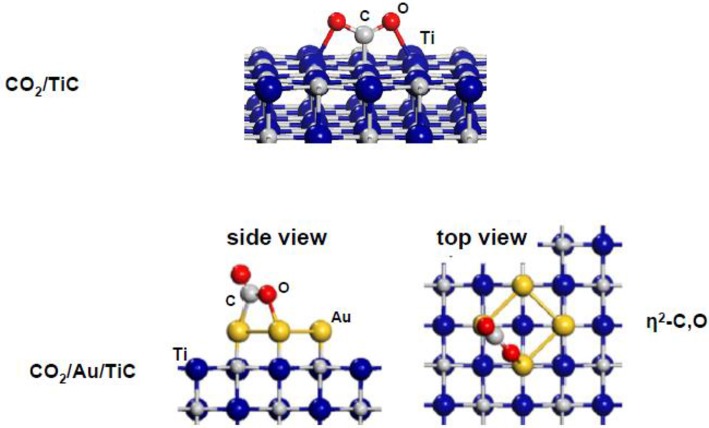
Adsorption geometries obtained with DF calculations for CO_2_ on TiC(001) and Au_4_/TiC(001) surfaces. Reproduced with permission from Vidal et al. (2012), copyright 2012 by the American Chemical Society of Chemistry.

Figure 11 shows results of DF calculations for the bonding geometry of the CO_2_ molecule on plain TiC(001) (Vidal et al., 2012). The molecule binds in a seudo-η^3^-C,O,O configuration with one single C-C bond (1.48 Å in length) and two weak Ti-O bonds (2.24 Å in length). A net carbide → CO_2_ electron transfer leads to activation and bending of the CO_2_ molecule on TiC(001). The CO_2_ adsorption process induces an elongation of the C-O bonds from 1.17 Å in the free molecule (gas phase) to 1.29 Å on the carbide surface. An adsorption energy of −0.62 eV was calculated for the CO_2_ on TiC(001). This is a moderate value for a binding energy but in magnitude it is still much larger than adsorption energies found in experimental and theoretical studies for CO_2_ interacting with surfaces of copper and late transition metals (Freund and Messmer, 1986; Freund and Roberts, 1996; Taifan et al., 2016). Substantial binding energies have also been calculated for CO_2_ on other MC(001) surfaces with the molecule mainly interacting with C sites of the carbide (Vidal et al., 2012; Posada-Perez et al., 2014; Posada-Pérez et al., 2016; Kunkel et al., 2016). On carbide surfaces, an extremely strong interaction has been observed for CO_2_ on β-Mo_2_C(001) (Ren et al., 2006; Posada-Perez et al., 2014), where the low C/metal ratio leads to CO2➜CO➜C transformations below room temperature and DF calculations show an almost spontaneous cleavage of the first C-O bond (Posada-Perez et al., 2014).

The trends observed in theoretical studies (Yang et al., 2013; Posada-Pérez et al., 2016) indicate that a 1:1 carbon-to-metal ratio is the best option if one is interested in the conversion of CO_2_ to oxygenates and wants to reduce methane formation. The addition of small gold particles to TiC(001) and MoC(001) surfaces produces systems with remarkable activity for the hydrogenation of CO_2_ to methanol (Vidal et al., 2012; Posada-Pérez et al., 2016). At the bottom of Figure 11, one can see the calculated geometry for the binding of CO_2_ to a Au_4_/TiC(001) system (Vidal et al., 2012). The CO_2_ binding energy over Au_4_/TiC(001) was substantial, −0.68 eV, and the molecule was attached to the gold atoms with a η^2^-C,O conformation. In contrast, the binding energy of CO_2_ on extended surfaces of metallic gold or unsupported (i.e., free) gold nanoparticles is zero (Freund and Messmer, 1986; Freund and Roberts, 1996; Vidal et al., 2012). On Au_4_/TiC(001), the molecule exhibits the bended geometry of a charged species (Vidal et al., 2012) with a significant elongation (0.05–0.11 Å) of the C-O bonds with respect to the calculated value in gas phase (1.17 Å, see above) (Vidal et al., 2012). Thus, one can conclude that a Au_4_/TiC(001) surface clearly activates the CO_2_ molecule.

**Figure 12:**
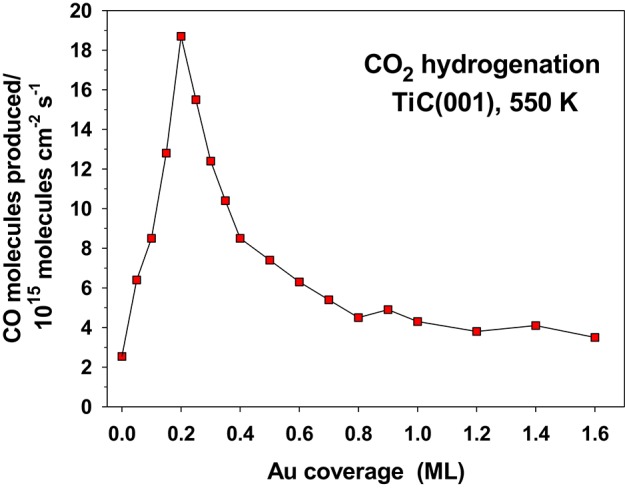
Rate for the yield of CO during the hydrogenation of CO_2_ on a series of Au/TiC(001) surfaces. T = 550 K, P_CO2_ = 0.5 atm, P_H2_ = 4.5 atm. Reproduced with permission from Posada-Pérez et al. (2016), copyright 2016 by the American Chemical Society.

The hydrogenation of CO_2_ on Au/TiC(001) and Au/MoC yields CO, the main reaction product, and methanol (Vidal et al., 2012; Posada-Pérez et al., 2016). The amount of CO produced is 2–3 orders of magnitude bigger than the yield of methanol. The amount of gold deposited on the carbide surface has a very strong effect on the activity of the system, Figure 12. An optimum performance was found at Au coverages of 0.1–0.2 ML, when there was a large amount of small 2D clusters on the carbide substrates (Vidal et al., 2012; Posada-Pérez et al., 2016). Large 3D clusters of Au display a low activity for CO_2_ hydrogenation pointing to the need of electronic perturbations in the Au adatoms. In Figure 11, a flat Au_4_ cluster binds the CO_2_ molecule well, but when the Au_4_ is replaced by a 13-atom pyramid of gold (see Figure 1), there is no binding of the CO_2_ molecule (Vidal et al., 2012; Posada-Pérez et al., 2016).

**Figure 13:**
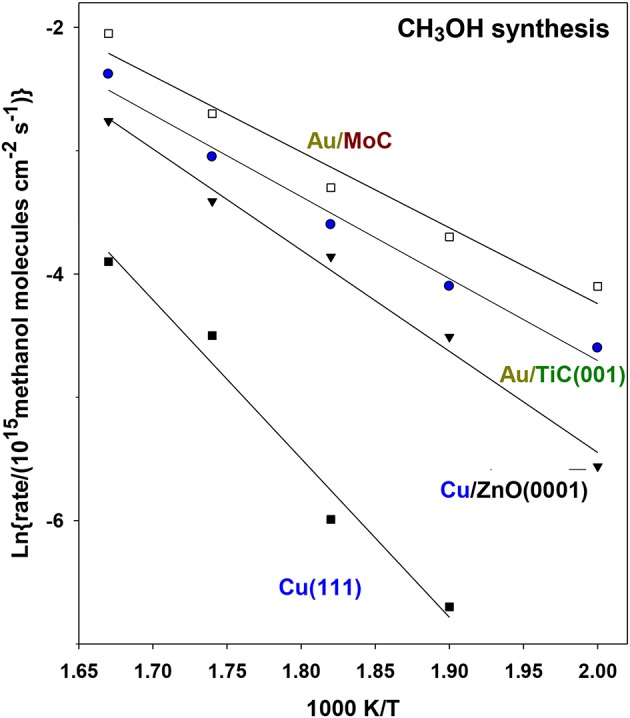
Arrhenius plots for the generation of CH_3_OH through CO_2_ hydrogenation on a several Au-containing catalysts. Initially, 0.2 ML of gold were deposited on MoC and TiC(001). In a batch reactor, both catalysts were exposed to 0.049 MPa (0.5 atm) of CO_2_ and 0.441 MPa (4.5 atm) of H_2_ at temperatures of 600, 575, 550, 525, and 500 K. Reproduced with permission from Posada-Pérez et al. (2016), copyright 2016 by the American Chemical Society.

Figure 13 displays Arrhenius plots for the generation of CH_3_OH on Au/TiC(001) and Au/MoC (Vidal et al., 2012; Posada-Pérez et al., 2016). For comparison are included results for Cu(111) and a system which models an industrial Cu/ZnO catalyst for methanol synthesis. The calculated apparent activation energies for methanol and CO production are listed in Table 1. In the case of methanol synthesis, the apparent activation energy drops from a value of 25 Kcal/mol on Cu(111) to 13 and 12 kcal/mol for Au supported over TiC(001) and MoC (Vidal et al., 2012; Posada-Pérez et al., 2016). These surfaces exhibited a methanol production rate that was 8–11 times larger than that seen for Cu/ZnO(000i), Figure 13, illustrating the great advantage of using a carbide as a metal support (Vidal et al., 2012; Posada-Pérez et al., 2016). In Table 1 are listed apparent activation energies for CH_3_OH and CO formation on different catalysts. For a given surface, one can see similar values for CH_3_OH and CO formation hinting that CO production is the rate limiting step on all the metal/carbide surfaces. Thus, CO is probably formed first, through the reverse WGS reaction, and a fraction of the formed CO is further converted into methanol via selective hydrogenation steps (Vidal et al., 2012; Posada-Pérez et al., 2016).

**Table 1:** Apparent activation energies for CO_2_ hydrogenation on a series of (in kcal/mol)^a^.

**Catalyst**	**CO, RWGS**	**CH_**3**_OH synthesis**
Au/δ-MoC	10	12
Au/TiC(001)	14	13
δ-MoC	18	17
TiC(001)	19	21
Cu/ZnO(000i)	14	16
Cu(111)	22	25

a *From Posada-Pérez et al. (2016)*.

**Figure 14:**
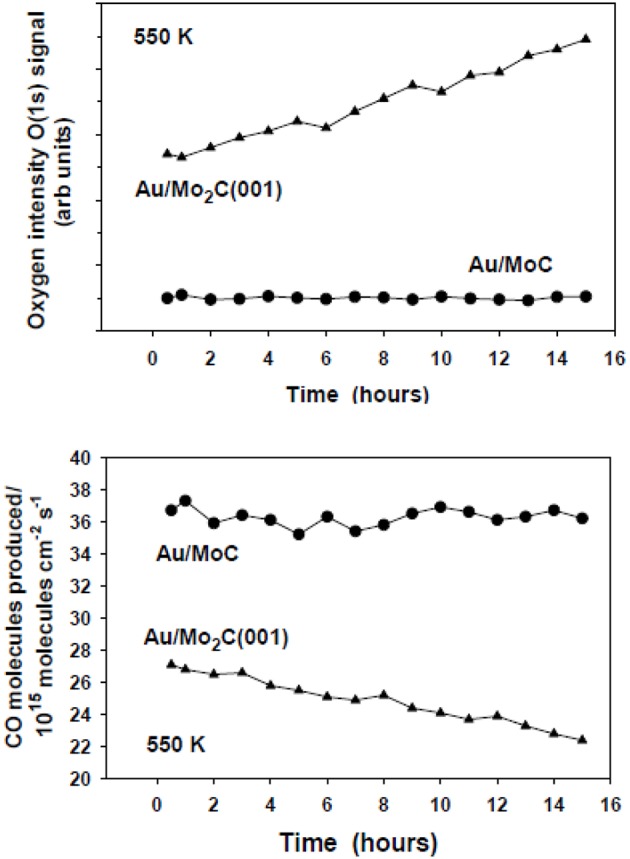
Top: Coverages of O measured with XPS for Au/β-Mo_2_C(001) and Au/δ-MoC catalysts (θ_Au_ ~0.2 ML) as a function of time under constant CO_2_ hydrogenation conditions. Bottom: Corresponding rate of CO generation over the Au/β-Mo_2_C(001) and Au/δ-MoC catalysts as a function of time maintaining the same reaction mixture. In a batch reactor, both catalysts were exposed to 0.049 MPa (0.5 atm) of CO_2_ and 0.441 MPa (4.5 atm) of H_2_ at a temperature of 550 K. Reproduced with permission from Posada-Pérez et al. (2016), copyright 2016 by the American Chemical Society.

After reaction, the existence of a minor coverage of oxygen (~0.1 ML) was detected with XPS over the TiC(001) and MoC substrates (Vidal et al., 2012; Posada-Pérez et al., 2016). The amount of oxygen present on these carbide catalysts did not increase with time producing a decrease in catalytic activity (see Figure 14). A completely opposite behavior was seen for Au/β-Mo_2_C(001) where the amount of oxygen present on the surface was substantial (>0.4 ML) and raised with time (Figure 14) probably as a consequence of the formation of an oxycarbide. Therefore, the Au/β-Mo_2_C(001) catalyst displayed poor stability because the surface activity was reduced by O poisoning (Figure 14). These data point to the importance of the metal/carbon ratio in a transition metal carbide. It is a critical parameter to consider when aiming for a catalyst with good activity, selectivity, and stability for the hydrogenation of CO_2_ (Vidal et al., 2012; Posada-Pérez et al., 2016).

## Conclusion and Future Work

The experimental and theoretical results discussed above show that the electronic perturbations induced by the bonding of Au to a metal carbide have a strong impact on the performance of the noble metal in reactions associated with C1 catalysis such as the oxidation of CO, the production of hydrogen via the water-gas shift and the hydrogenation of CO_2_. On the carbide surfaces, the Au interacts stronger than on oxides opening the door for strong metal-support interactions.

So far, the experimental studies have been focused on a few reactions for Au particles supported on MoC and TiC. After studying the interaction of gold and several metal carbides with DF-based methods (Rodriguez and Illas, 2012), it is clear that the electronic perturbations on gold significantly rise when going from TiC to ZrC or TaC as a support. Thus, Au/ZrC and Au/TaC have the electronic properties necessary for being good catalysts and should be tested for C1 catalysis. Furthermore, the activity of the Au-carbide interfaces should be also tested for many of the reactions where catalytic activity has been observed on Au-oxide interfaces. For example, in the area of C1 chemistry, systematic studies must be carried out for CO-PROX (preferential CO oxidation), the hydrogenation of CO_2_ to CH_4_ or formic acid, or the reforming and manipulation of methanol.

## Author Contributions

The author confirms being the sole contributor of this work and has approved it for publication.

### Conflict of Interest

The author declares that the research was conducted in the absence of any commercial or financial relationships that could be construed as a potential conflict of interest.
